# Moving beyond traditional therapies: the role of nanomedicines in lung cancer

**DOI:** 10.3389/fphar.2024.1363346

**Published:** 2024-02-08

**Authors:** Jingjing Zhang, Yanzhi Li, Sa Guo, Weifen Zhang, Bing Fang, Shaohui Wang

**Affiliations:** ^1^ Medical College of Qingdao Binhai University, Qingdao, China; ^2^ The Affiliated Hospital of Qindao Binhai University (Qingdao Military-Cvil Integration Hospital), Qingdao, China; ^3^ Ethnic Medicine Academic Heritage Innovation Research Center, Meishan Traditional Chinese Medicine Hospital, Chengdu University of Traditional Chinese Medicine, Chengdu, China; ^4^ Medical College, Weifang University, Weifang, China

**Keywords:** lung cancer, nanomedicine, nanomedicine delivery system, passive targeting, active targeting

## Abstract

Amidst a global rise in lung cancer occurrences, conventional therapies continue to pose substantial side effects and possess notable toxicities while lacking specificity. Counteracting this, the incorporation of nanomedicines can notably enhance drug delivery at tumor sites, extend a drug’s half-life and mitigate inadvertent toxic and adverse impacts on healthy tissues, substantially influencing lung cancer’s early detection and targeted therapy. Numerous studies signal that while the nano-characteristics of lung cancer nanomedicines play a pivotal role, further interplay with immune, photothermal, and genetic factors exist. This review posits that the progression towards multimodal combination therapies could potentially establish an efficacious platform for multimodal targeted lung cancer treatments. Current nanomedicines split into active and passive targeting. Active therapies focus on a single target, often with unsatisfactory results. Yet, developing combination systems targeting multiple sites could chart new paths in lung cancer therapy. Conversely, low drug delivery rates limit passive therapies. Utilizing the EPR effect to bind specific ligands on nanoparticles to tumor cell receptors might create a new regime combining active-passive targeting, potentially elevating the nanomedicines’ concentration at target sites. This review collates recent advancements through the lens of nanomedicine’s attributes for lung cancer therapeutics, the novel carrier classifications, targeted therapeutic modalities and their mechanisms, proposing that the emergence of multi-target nanocomposite therapeutics, combined active-passive targeting therapies and multimodal combined treatments will pioneer novel approaches and tools for future lung cancer clinical therapies.

## 1 Introduction

Lung cancer, presently, stands as the leading cancer system around the globe and in China, maintaining an alarmingly high mortality rate that prompts imminent and novel medical interventions (The, 2019; Bade and Dela Cruz, 2020). In a world dismayed by the repercussions of the deadliest disease, it is remarkable to acknowledge the pathological intricacies of this prevalent malady. Lung cancer is broadly classified into two overarching categories. The first, Small Cell Lung Cancer (SCLC), encapsulates 15%–20% of the overall occurrence. Meanwhile, the non-Small Cell Lung Cancer (NSCLC) predominantly accounts for the remaining cases, marking it as a dominant contributor in the overall lung cancer statisticon (Salehi-Rad et al., 2020; Xie et al., 2021). The clinical landscape in the realm of lung cancer treatment offers a variety of therapy methods with assorted degrees of effectiveness. Surgical excision, radiotherapy, chemotherapy, molecular targeted therapy, and immunotherapy count amongst the more recognizable treatment modalities. The primary empirically prevalent treatment strategy, however, remains chemotherapy, implemented in more than 90% of lung cancer cases. Yet, despite its widespread incorporation, the limitations associated with chemotherapy have yet to be thoroughly surmounted. Reflecting on the current clinical therapy, a discernible shadow looms large over chemotherapy due to significant drawbacks (Hirsch et al., 2017; Miller and Hanna, 2021; Wang et al., 2021). Examples of such shortcomings are rendered manifestations by the effects of drugs like paclitaxel and adriamycin, noted for their potent and toxic side effects (Jain and Thareja, 2019). These medications have been implicated in inducing drug-resistant mutations in a multitude of tumors, leading to escalation in the complexity of lung cancer therapy. Such factors invariably contribute to the challenges experienced in this field. In addition to the aforementioned, conventional drugs employed for lung cancer therapy are frequently recognized for their low solubility, poor stability, lack of specificity, high resistance, and toxicity. These standing issues, collectively, affect the clinical applicability of these drugs on variable scales, constituting eminent roadblocks towards an ideal therapy method for lung cancer (Crintea et al., 2021). As a beacon of hope in this daunting situation, modern scientific breakthroughs emerge from the horizons of research, unveiling potential solutions. Several contemporary studies have underscored the potential of nanomedicines as a promising alternative, possessing the ability to revolutionize the paradigm of lung cancer treatment.

Nanomedicines, touted as the new age therapeutic intervention, are unearthed as powerful tools to confront the traditional limitations of chemotherapeutic drugs. Their exceptional attributes that include high specificity, minimization of systemic toxicity, and improved water solubility are hard to overstate (Gadekar et al., 2021). Furthermore, their ability to target tumor tissues specifically is a characteristic that has magnetized the attention of researchers worldwide (Guo et al., 2020). Consequently, nanomedicines have ascended to the limelight in research circles, promising significant advances in lung cancer therapy. Despite the mammoth-like challenges posed by lung cancer, the ascendance of these diminutive nanomedicines offers a glimpse of hope for a brighter future.

This comprehensive analysis synthesizes the latest progress in the context of nanomedicine’s properties for the treatment of lung cancer. It offers an in-depth exploration of innovative carrier classifications and targeted therapeutic modalities, inclusive of their operating mechanisms. This synthesis advances the notion that the pioneering field of multi-target nanocomposite therapeutics, amalgamated active-passive targeting treatments, as well as multifaceted combined therapies, could initiate groundbreaking strategies in the realm of lung cancer clinical treatment for future applications. By envisaging these emerging perspectives, this evaluation strives to outline the future course of lung cancer therapeutics amidst dynamically evolving nanomedical frontiers.

## 2 The innovation of nanomedicines in cancer treatment

Nanomedicines employ state-of-the-art nano preparation methodologies to fashion drugs and other particulate entities in nanometric dimensions. Alternatively, they may incorporate suitable carrier materials with drugs to generate nanoparticles scaling to nanometer dimensions. Conventional anti-cancer nanomedicines delineate particle dimensions specifically within the range of 1–200 nm (Doroudian et al., 2021a). This highly specific size range provides these minute particles with extraordinary capabilities. Contrasting with mainstream drugs, nanomedicines amplify solubility and absorption rates of otherwise insoluble chemotherapeutic agents, thereby minimizing the necessary drug dosage. This salient feature encompasses the potency to transform the therapeutic landscape significantly by curbing potentially harmful dosage amounts. Simultaneously, nanomedicines hold the capacity to bestow drugs with newfound characteristics, shifting the balance in favour of more efficacious and compatible chemotherapeutic experiences. These pioneering properties range from a reduction of detrimental side effects to an amplification of biocompatibility, promoting a more patient-friendly approach to therapeutic regimens. Furthermore, nanomedicines can extend the half-lives of drug particles delivered to target organs (Markman et al., 2013), ensuring a more sustained, controlled release, and thereby improving the effectiveness of therapeutic interventions (Liu et al., 2022). In another remarkable twist, certain variants of nanoparticles (NPs) demonstrate unparalleled multifunctional capabilities that transcend basic medical applications. These specialized particles provide a gateway to facilitate tumor imaging and diagnostic procedures while simultaneously operationalizing therapeutic strategies. Upholding a comprehensive approach, they hold great potential to introduce multidisciplinary biomedical applications in the treatment of lung cancer (Mukherjee et al., 2019; Zhao et al., 2023). This thereby, emphasizes the pioneering role of nanomedicines, placing them on the frontline of innovative lung cancer therapeutic regimes. Currently, nanotherapeutics for targeted lung cancer therapy are shown in Table 1.

**TABLE 1:** Nanotherapeutics for targeted lung cancer therapy.

Nanotherapeutic name/trade name	Targeting agent	Therapeutic agent	Ref.
Docetaxel (DTX)-loaded hyaluronic acid (HA) nanocapsules	HA	DTX	Maiolino et al. (2015)
DTX-HPLGA	HA	DTX	Wu et al. (2017)
DTX-HACTNPs	HA	PTX	Wu et al. (2017)
DOX/HA-ss-DOX micelles	HA	DOX	Yin et al. (2018)
DTX/PPN@PPY@HA	HA	DTX	Zhao et al. (2019)
cRGD-PLGA@DOX	cRGD	DOX	Huang et al. (2020)
FA/Tf-CDDP-NPs	Folic acid, Transferrin	Cisplatin	Tan and Wang (2018)
LPs-DTX-FA	Folic acid	DTX	AlSawaftah et al. (2021)
Tf-SS-Afa-LPNs	Transferrin	Afatinib	Kumari et al. (2022)
Au-siRNA-PAA-AS1411	AS1411 aptamer	DOX, siRNA	Yang et al. (2021)

Note: DTX-HPLGA: hyaluronic acid (HA) coated PLGA, nanoparticulate docetaxel; DTX-HACTNPs: HA, coupled chitosan nanoparticles bearing docetaxel; DOX/HA-ss-DOX, micelles: Free adriamycin-loaded pH/reduction dual-responsive hyaluronic acid-adriamycin prodrug micelles; DTX/PPN@PPY@HA: Docetaxel-loaded polypyrrole (PPY) and HA-modified phospholipid nanoparticles; cRGD-PLGA@DOX: Doxorubicin (DOX) encapsulated by a cyclic arginine-glycine-aspartic acid polypeptide modified poly-(lactic-co-glycolic acid) nanosystem; FA/Tf-CDDP-NPs: Folic acid (FA) and transferrin (Tf) modified cisplatin (CDDP) loaded nanoparticles; LPs-DTX-FA: Docetaxel-loaded folic acid-conjugated liposomes; Tf-SS-Afa-LPNs: Afatinib loaded, Tf modified redox-sensitive lipid-polymer hybrid nanoparticles; Au-siRNA-PAA-AS1411: Au-siRNA@ aptamer nanocages.

### 2.1 Properties and characteristics of nanomedicine

Consequent to the accelerated proliferation of cells in the tumor vicinity and the aberrant generation of new blood vessels, there is an astronomical surge in oxygen consumption. This results in the formulation of an anoxic and mildly acidic environment in the tumor region, hampering the therapeutic efficacy of small-molecule chemotherapy drugs. Counteracting this challenge, nanomedicines leverage their unique size effect to permeate the space between tumor vascular endothelial cells via the Enhanced Permeability and Retention (EPR) effect indispensable to solid tumors. This enables them to agglomerate within tumor tissues, facilitating a higher number of drug molecules to reach the tumor tissues and be absorbed by tumorous cells. Consequently, the bioavailability of the drug molecules escalates significantly (Sindhwani et al., 2020). The nanometric size of these remedial interventions empowers them to optimally influence the clinical applicability of drugs. Hence, strategic nano sizing of the nanomedicines catalyzes the selective distribution of drugs within tumor tissues. This, in turn, mitigates the toxic side effects while amplifying the clinical therapeutic outcomes (Farjadian et al., 2019). Additionally, the utilization of nano-sized particles as carriers amplifies their efficiency to transport a variety of therapeutics, ranging from small molecules to peptides, proteins, and nucleic acids. Such nano carriers act dazzlingly to safeguard the small molecule drugs from the ambient environment, guarding them against recognition and inactivation by the immune system. This accentuates the bioavailability of the molecular drugs, reinforcing the drug’s desired therapeutic effect (Patra et al., 2018), which is instrumental in enhancing their effectiveness and achieving superior therapeutic outcomes.

### 2.2 Nanomedicine in lung cancer

The limitations associated with conventional lung cancer therapeutic methods are compelling a paradigm shift towards the innovative path of nanomedicines. Embedded within nanomedicines are properties that make them potentially transformative for the detection of lung cancer. The remarkable surface effect inherent in nanomedicines amplifies the binding efficiency of ligands to receptors overwhelmingly expressed in tumor cells. When deployed as contrast agents for lung cancer imaging, they have the potential to substantially elevate the sensitivity and specificity of diagnostic methods (Patra et al., 2018). Drawing on empirical evidence, Sun et al. (2019) successfully demonstrated that gold nanoparticles coated with a compound of ethylene glycol and chitosan (GC-AuNPs) serve as superb photoacoustic contrast agents for cancer cell imaging. Leveraging the beneficial physical, chemical, biological, and electrical properties of inorganic nanoparticle imaging contrast agents, diagnostic procedures such as Positron Emission Tomography (PET), Magnetic Resonance Imaging (MRI), and Single Photon Emission Computed Tomography (SPECT) can enhance their sensitivity. As a consequence, these methods can robustly monitor the progression of lung cancer in real-time during the course of therapy (Yin et al., 2021).

In addition to diagnostic applications, nanomedicines are also revolutionizing the therapeutic landscape for lung cancer. Nanoparticles (NPs), due to their unique characteristics, serve as effective vehicles for drug delivery, encapsulating or coupling with therapeutic agents and transporting them efficiently to tumor sites via controlled release. Drawing from their distinctive nanoscale size, high surface-area-to-volume ratio, and standout surface effects, they can modulate the pharmacokinetic and pharmacodynamic characteristics of anti-tumor drugs, thereby enhancing their curative efficacy. Further bolstering the promise of nanomedicines, polyethylene glycol-modified liposomes exhibit properties that shield them from recognition and phagocytosis by the mononuclear phagocytic system. This endows them with a prolonged circulation duration (García-Fernández et al., 2020), and amplifies their potential as therapeutically advantageous nano-agents. Hence, nanomedicines, underpinned by their grouped characteristics and unique properties, are leading a charge in the development and innovation of anti-cancer drugs, fundamentally reshaping the face of therapeutic interventions for lung cancer.

## 3 Nanomedicine carriers involved in the therapy of lung cancer

**FIGURE 1:**
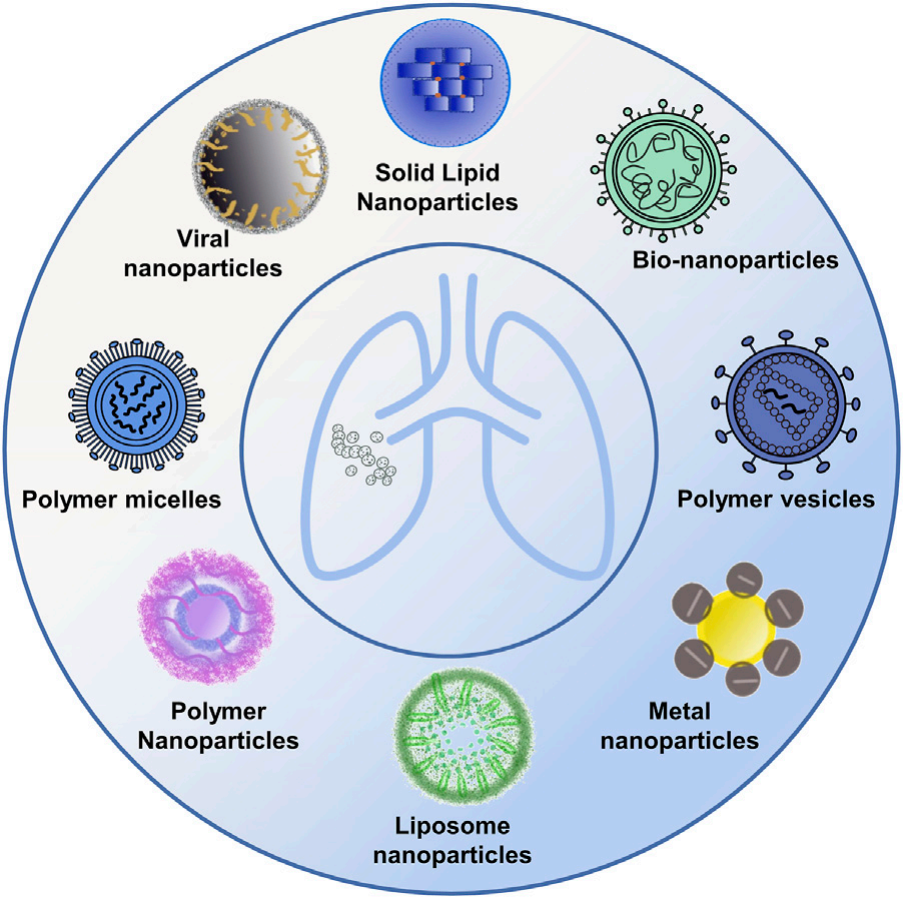
Diverse nanomedicine carriers. The figure presents a schematic representation showcasing a variety of nanocarrier designs utilized in drug delivery, including metal nanoparticles, polymeric nanoparticles, liposome nanoparticles, and bio-nanoparticles, among others.

Several studies have shed light on the significance of using nanoparticle carriers with diverse structures in creating antitumor drugs-an approach that has garnered noteworthy results in the clinical treatment of lung cancer. Current nanoparticle carriers tailored for lung cancer encompass a variety of forms, including metal nanoparticles, liposome nanoparticles, polymeric nanoparticles, and bio-nanoparticles, among others (Figure 1; Table 2).

**TABLE 2:** Lung cancer nanodrugs with different nanocarriers.

Nanomedicine	Therapeutic agent	Type of nanosized system	Action mechanism	Advantages	Ref.
Abraxane	Paclitaxel	Protein nanoparticles	Inhibits cell division by binding and stabilizing microtubules and preventing microtubule reorganization during cell division	Increased uptake and accumulation of paclitaxel in tumor tissues via gp-60-mediated endothelial cell transmembrane transport and protein SPARC interactions using the properties of albumin	Tan and Tan (2022)
BIND-014	Docetaxel	Polymeric nanoparticles	Inhibits the division and growth of tumor cells	Favorable drug encapsulation, prolonged circulation time and sustained drug release	Doroudian et al. (2021b)
XMT-1001	Camptothecin	Polymeric nanoparticles	Topoisomerase I to inhibit tumor cell growth and division	Improve drug stability and biocompatibility	Doroudian et al. (2021b)
Doxil	Doxorubicin hydrochloride	Liposomal nanoparticles	Topoisomerase II and DNA-dependent RNA polymerase activity and generation of oxygen radicals leading to DNA damage and cell death	Liposome-encapsulated adriamycin effectively reduces its toxicity and prolongs the drug’s residence time in the bloodstream	Rahim et al. (2021)
DTX-loaded PLGA@Au Nanoparticles	Docetaxel	Metal nanoparticles	Sustained drug release by controlling the degradation rate of PLGA nanoparticles	Utilizing the light-absorbing and light-scattering properties of gold nanoparticles can also serve a variety of roles, such as photothermal therapy and enhanced photothermal effects	Hao et al. (2015)

### 3.1 Metal nanoparticles

**FIGURE 2:**
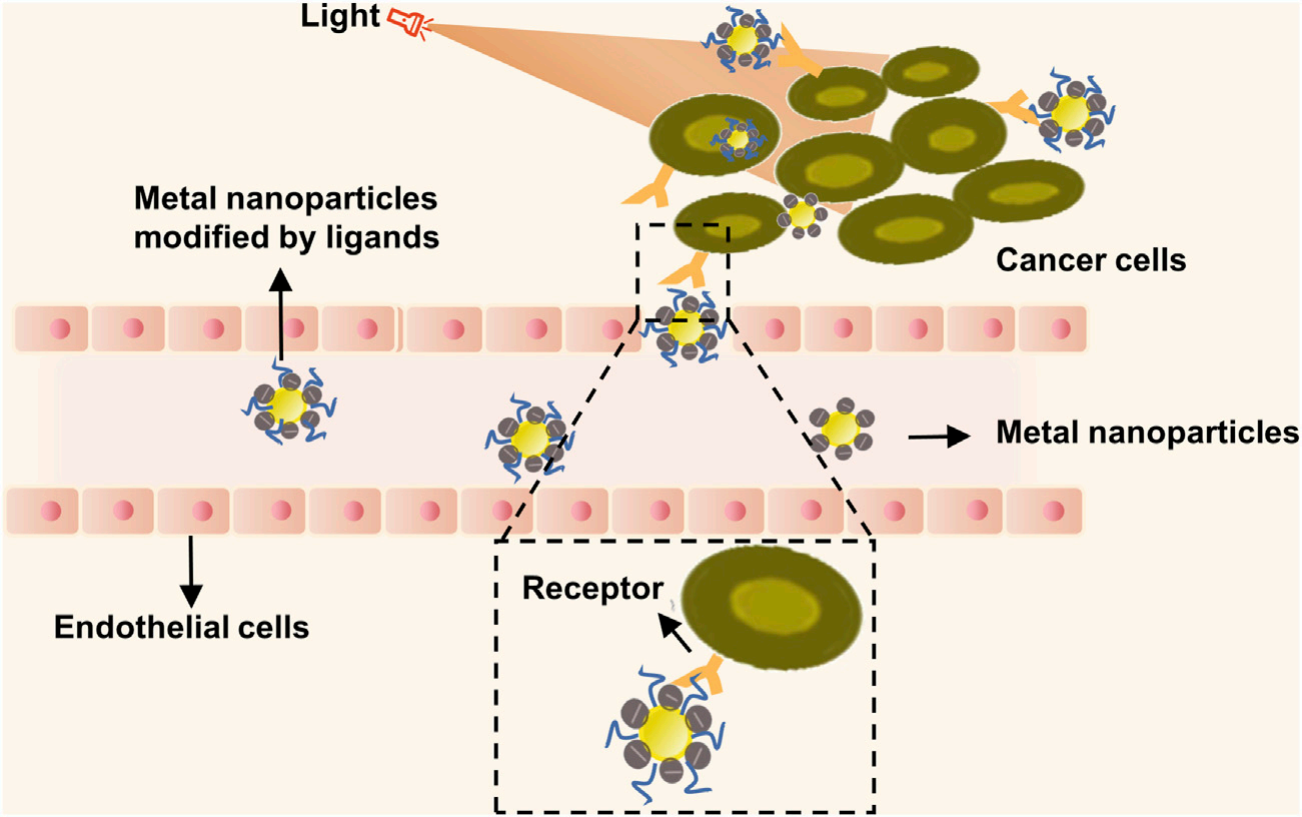
Metal nanoparticles targeted therapy combined with photothermal therapy for tumor therapy. Metal nanoparticles containing numerous mobile electrons can absorb energy from an external light source. Subsequently, they generate significant heat energy and reverse the direction of heat loss, resulting in hyperthermia from the inside out. This phenomenon induces localized thermal destruction, leading to cell lysis, enzyme release, and ultimately, cell necrosis and protein degeneration.

Several studies illustrate how metal nanoparticles function as dense, spherical carriers, constituting the smallest class of nanoparticles. These can be engineered from pure metals such as gold, silver, and iron, or non-metal heteromorphs (Mauro et al., 2021). While these nanoparticles cannot encapsulate drug molecules, they do have the ability to couple with drug molecule surfaces, enabling targeted therapy for tumor cells (Guido et al., 2022). Metal nanoparticles showcase localized surface plasmon resonance (SPR), electrochemical properties, unique surface traits for easy modification, and biocompatibility. These nanoparticles, once surface-modified, can be linked to target drugs or structures through either covalent or non-covalent bonding, culminating in multi-functional nanoparticles geared towards tumor imaging, phototherapy, and photo modulation applications (Figure 2). In photothermal therapy (PTT), nanoparticles or other photothermal agents are employed to absorb near-infrared (NIR) light, leading to a localized temperature increase that is sufficient to ablate cancer cells while minimizing damage to surrounding tissues. Upon NIR irradiation, the absorbed energy is converted into heat, elevating the temperature to the range of 42–47°C within the tumor microenvironment. This targeted thermal effect induces cellular destruction primarily via apoptosis, offering a minimally invasive treatment option that can be selectively applied to various types of solid tumors. Given its precision and comparatively low side effects, PTT has emerged as a promising modality in the field of oncology, capable of overcoming certain limitations associated with conventional therapies (Zhi et al., 2020; Tang et al., 2023). A noteworthy revelation confirms that pairing metal nanoparticles with specific antibodies, through a signal amplification pathway, can effectively detect tumor markers present in circulating blood. This significantly boosts lung cancer detection rates and expedites early diagnoses (Singh et al., 2018). In a study by Ashton et al., polyethylene glycol gold nanoparticles were used as a contrast agent for CT imaging of lung cancer in a mouse model. Their findings suggest that these nanoparticles considerably enhance image contrast and offer unique targeting properties, bearing much importance in the realms of tumor imaging and targeted diagnosis (Ashton et al., 2014). Moreover, metal nanoparticles can convert absorbed photons into thermal energy, invoking a photothermal solid effect which further augments the therapeutic impact (Patra et al., 2018). Exploring further, Yu et al. used an organic semiconductor pre-nano stimulant (OSPS), developed by them, in tumor therapy. Their study displayed how combining this OSPS with photothermal and immune effects could structure a photothermal drug delivery system capable of controlled drug release and enhanced antitumor immune responses. Thus, research on metal nanoparticles emphasizes their nano-effect, photothermal effects, and immune effects, giving rise to multi-modal combined therapy strategies that could have immense implications in the imaging, identification, and treatment of lung cancer (Yu et al., 2021).

### 3.2 Polymeric nanoparticles

Polymeric nanoparticles act as capsulated drug carriers, often employed for hydrophobic drug delivery, due to their robust capacity, minute size, expansive surface area, and notable biofilm adhesion properties that allow for local retention (Ahlawat et al., 2018). The core-shell structure of these nanoparticles enables the encapsulation of hydrophobic drugs, while extending circulation times and drug release periods. Additionally, their surfaces can be tailored with ligands for targeted drug delivery (Mukherjee et al., 2019). Reflecting on a significant milestone in 2005, Celgene, an American biopharmaceutical company, successfully developed albumin-bound paclitaxel, receiving FDA approval for treating non-small cell lung cancer (Kundranda and Niu, 2015). In subsequent *in vitro* cloning experiments with A549 cells, researchers combined polymeric nanoparticles with radiotherapy and compared their effect with paclitaxel alone. Results revealed polymeric nanoparticles are able to uphold the antitumor activity of free paclitaxel (Jung et al., 2012). In another study, Jiang et al. (2015) underlined a substantial cytotoxic influence of crizotinib (CZT) on NCI-H3122 lung cancer cells, demonstrating a dose-dependent effect and thus reflecting the superior anticancer capacity of polymeric nanoparticles (Kwon et al., 2019). Observations revealed that these nanoparticles distributed within cytoplasmic regions, showcasing a traditional endocytosis-mediated cellular uptake. However, it is crucial to note that polymeric nanoparticles exhibit instability and are prone to hydrolysis, which could lead to automatic oxidation and reduction in film fluidity. There’s a possibility of encountering drug percolation, aggregation, and precipitation, due to these characteristics.

### 3.3 Liposomal nanoparticles

Liposomes represent lipid vesicles surrounded by cellular membrane-like structures and comprise phospholipid bilayers. These bilayers can effectively encapsulate drug molecules and subsequently deliver those to targeted tumor cells. They can accommodate both hydrophilic and lipophilic drugs and can be categorised as trigger-release liposomes or drug-carrying liposomes (Doroudian et al., 2021a). Liposomes are characterised by commendable biocompatibility and are able to adjust the distribution of their encapsulated drug molecules by slowing down clearance rates and extending intra-vascular circulation durations (Mauro et al., 2021). This makes them ideally suited as drug delivery system carriers, especially for antitumor drug delivery, enhancing the therapeutic efficacy of such drugs. A noteworthy milestone was the approval of azithromycin nano-formulations developed by Barenholz by the FDA in 1995. They projected that the nano-liposomes could be used for drug leakage from tumor vasculature into tumor tissues using the EPR effect (Barenholz, 2012). Bakhtiary et al. encapsulated erlotinib using solid lipid nanoparticles and employed a dry powder inhaler for its administration. They reported an enhancement in the stability between hydrophobic and hydrophilic molecules, the avoidance of organic solvents, and an improvement in the drug’s pharmacokinetic profile (García-Fernández et al., 2020). Eboa et al., in their study involving a mouse model of lipopolysaccharide-induced endotoxic shock, showed that the delivery of cobicistat via bilayer cross-linked multilayer lipid vesicles could effectively inhibit the formation of neutrophil extracellular trap networks (NETs), thereby suppressing the production of neutrophil elastase (NE) and other pro-inflammatory factors while mitigating lung injuries in mice (Okeke et al., 2020). The recent significant advancements in mRNA vaccines centred on liposome nanoparticles as carriers in tumor immunotherapy is propelling research to examine combined applications of liposome drugs and immune therapeutic agents (Wang et al., 2022). The review also revealed that liposomes have the ability to induce cells to produce robust responses to various cytokines, bolster the phagocytic capability of macrophages, and decrease the level of inflammatory mediators. This, in turn, can enhance lymphocyte proliferation and differentiation, thereby boosting immune function. These findings reinforce the conclusion that liposomes exhibit excellent tumor permeability and inhibitory abilities against tumor growth. By serving as nanomedicine carriers in tumor immunotherapy and enabling the specific accumulation of drugs at tumor sites while promoting the uptake of these drugs by tumor cells, the applications of liposome nanoparticles are vast.

### 3.4 Bio-nanoparticles

Bio-nanoparticles have drawn significant scientific attention due to their commendable biocompatibility, robust stability, and biodegradability. Types of biological nanoparticles encompass protein, viral, aptamer, and iron-loaded protein particles (Mukherjee et al., 2019).

### 3.5 Viral nanoparticles

Viral nanoparticles (VNPs), derived from viruses and phages, play critical roles in drug delivery, biosensing, bioimaging, and vaccine development. Their merit lies in their superior biocompatibility, size and shape versatility, and the ease with which their surfaces can be modified (Mukherjee et al., 2019). Targeted molecules congregated on a virus’s surface can multivalently bind to receptors, thereby augmenting the uptake of these molecules by tumour cells. Nanoconstructs with specific recognitive capabilities can be created by attaching them onto biomaterials, employing viral adsorption or mediated immune response mechanisms. Viruses can also serve as nano scaffolds, enabling multivalent linking of functional ligands at specific sites, thus demonstrating extensive applicability in sensing and imaging fields (Li et al., 2010). Building on this, Hu et al. loaded the prodrug 6-propionate Doxorubicin (DOX-EMCH) onto the empty core virus-like particles (VLPs) of the plant mottled virus (PhMV). They anchored doxorubicin molecules interactively by chemically binding cysteine residues and engaging in π-π stacking, and further coated the outer surface with polyethylene glycol (PEG) to enhance biocompatibility. This innovative approach culminated in a drug delivery system that exhibited stable and highly effective therapeutic outcomes (Hu and Steinmetz, 2020).

### 3.6 Protein nanoparticles

Protein nanoparticles, derived from natural proteins such as gelatin, gliadin, albumin, and soy protein, have gained attention for their potential use in drug and gene delivery. These nanoparticles possess desirable characteristics including high biocompatibility, easy degradation, and low or non-toxicity, addressing some of the limitations associated with inorganic nanocarriers. Moreover, protein nanocarriers offer various advantages, such as high drug binding capacity and the ability to target tumors using specific ligands (Zaher et al., 2023). The presence of surface functional groups on protein nanoparticles allows for easy modification. Additionally, the hollow structure of certain proteins enables the loading of small molecule drugs or metal nanoparticles, facilitating drug delivery and combination therapy. Sepehri et al. (Miao et al., 2022) demonstrated the construction of a hybrid of human serum albumin (HSA) and 7-ethyl-10-hydroxycamptothecin, an anticancer drug, through a reaction involving N-(3-dimethylaminopropyl)-N′-ethylcarbodiimide hydrochloride (EDC) and N-hydroxysuccinimide (NHS). The experimental results showed that the hybrid improved the drug’s solubility and pharmacokinetic profile.

Among them, ferritin is an iron storage protein that is widely distributed in the body. It is composed of self-assembled hollow protein nanocages consisting of 24 individual subunits. These nanocages have inner and outer diameters of 8 nm and 12 nm, respectively. They can be specifically targeted to tumor cells through the transferrin receptor 1. This unique structure allows ferritin to be loaded with anticancer drugs (Shesh and Connor, 2023). When ferritin binds to the transferrin receptor, it is taken up by tumor tissues, making it an effective vehicle for drug delivery. Furthermore, ferritin is highly stable, minimizing the amount of drug released during transportation. By taking advantage of ferritin’s tumor-targeting properties, drug carriers based on ferritin can significantly reduce the side effects of chemotherapeutic drugs. They achieve this by specifically targeting tumor cells for internalization and drug release in lysosomes. To extend the circulation time *in vivo*, the surface of ferritin can be modified with PEG (polyethylene glycol) (Miao et al., 2022). However, the efficacy of ferritin drug carriers that solely target TfR1 is limited due to differences in TfR1 expression levels on different tumor cells. To address this limitation, researchers have developed dual-targeting ferritin drug carriers. For example, Deng et al. (2023) genetically engineered a membrane-penetrating peptide called tLyP-1 and fused it to the N-terminal end of human ferrous ferritin (HFn). They loaded paclitaxel (PTX) into the HFn nanocage and demonstrated that this modified ferritin with tLyP-1 has dual receptor-mediated targeting function. It can effectively transport drugs to deeper tumor regions, overcoming the problem of insufficient drug delivery in solid tumors.

### 3.7 Iron-loaded protein nanoparticles

Ferritin, a protein that possesses a detoxifying factor, prevents the formation of reactive oxygen species by converting ferrous ions into iron’s insoluble form. Without an iron core, ferritin forms a hollow protein nanocage comprised of 24 self-assembled polypeptide subunits. Its inner and outer diameters measure approximately 8 nm and 12 nm, respectively (Dostalova et al., 2017). As a naturally occurring nanocarrier, Ferritin boasts a reliable drug release mechanism capable of accurately delivering drugs to tumor cells. Li et al. successfully synthesized a multifunctional hybrid nanostructure of ferritin, which exhibited green fluorescence. They accomplished this by embedding ferrous magnetic iron oxide nanoparticles in the inner cavity of ferritin, underlining its potential utility in tumor cell imaging and detection (Li et al., 2012).

## 4 Targeted therapy of lung cancer with nanomedicine

Targeted nanomedicine delivery systems have the potential to convey therapeutic drugs specifically to intended organs, thereby enhancing therapeutic efficacy and mitigating toxic side effects. Depending on the targeting mechanisms utilized, these delivery systems can be divided into two primary categories: passive targeting and active targeting. Passive targeting largely relies on the Enhanced Permeability and Retention (EPR) effect. Given the profuse vasculature surrounding tumors, nanomedicines can leverage the hyperpermeable cells and impaired lymphatic drainage to passively reach solid tumors, thereby promoting the selective drug distribution at the tumor site (Baetke et al., 2015). In contrast, active targeting typically involves the use of high-affinity ligands attached to the surface of nanodrugs which can specifically connect to receptors on the surface of tumor cells. This allows drugs to bind to targeted tumor cells and heighten the concentration of drugs at the tumor site (Rahim et al., 2021). Diverse ligands, including folic acid, antibodies, peptides, small molecules, and aptamers, have been developed for this purpose. Of notable importance, active targeting, based on the cellular transcytosis effect, offers an advantage over passive targeting that relies on the EPR effect. However, the molecular and cellular mechanisms involved in the active targeting of nanomedicines into solid tumors warrant further in-depth investigation. Hence, using the EPR effect, a novel therapeutic modality of both passive and active targeting can be introduced by combining the specific ligand on the surface of nanoparticles with the highly expressed receptor of tumor cells. This is expected to boost the uptake of drugs by tumor cells, ultimately improving the therapeutic impact on lung cancer (Table 3).

**TABLE 3:** Active and passive targeted nanomedicines.

Nanomedicine	Drug delivery system	Targeting	Reason	Ref.
Taxotere	Liposomal nanoparticles	Active and passive	Inhibits tumor growth by binding and stabilizing microtubule proteins and preventing mitosis of tumor cells	Mikhail et al. (2013)
CRLX101	Liposomal nanoparticles	Active	Inhibits tumor growth and spread by targeting receptors on the surface of tumor cells, entering the cells, and releasing drug molecules	Svenson et al. (2011)
BIND-014	Polymeric nanoparticles	Active	Nanoparticles loaded with polyene paclitaxel and specifically targeting PSMA	Von Hoff et al. (2016)
SGT-53	Liposomal nanoparticles	Active	TfRscFv modifies liposome nanocomplexes for p53 gene delivery and actively targets transferrin receptors in tumor cells	Senzer et al. (2013)
Doxil	Liposomal nanoparticles	Passive	Increasing the half-life and altering the distribution of drugs in the bloodstream exploits the properties of liposomes, vascular leakage, and lymphatic drainage to achieve drug delivery	Barenholz (2012)
Daunoxome	Liposomal nanoparticles	Passive	Using the size and surface properties of nanoparticles to increase their residence time in tumor tissue and release daunorubicin, thereby increasing the concentration and efficacy of the drug in tumor tissue	Guaglianone et al. (1994)
Abraxane	Protein nanoparticles	Passive	Exploiting the physical and chemical properties of paclitaxel-protein bound nanoparticles to enable more efficient drug accumulation in tumor tissue and less damage to healthy tissue	Miele et al. (2009)
NBTXR3	HfO_2_	Passive	Utilizing nanocrystals can increase the efficacy of radiation therapy by absorbing radiation and converting it into high-energy electrons and excited oxygen radicals	Zhang et al. (2021)
Docetaxel-PNP	Polymeric nanoparticles	Passive	Accumulation of drugs in target tissues by altering drug properties such as solubility, release rate and drug stability	Wang et al. (2014)

### 4.1 Passive targeting

**FIGURE 3:**
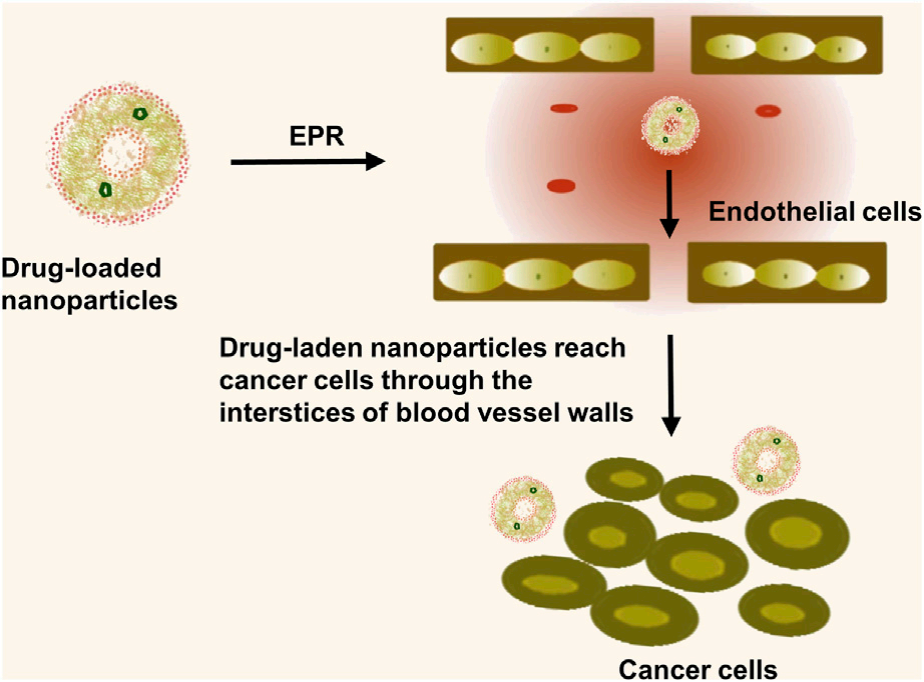
Passive targeting is the entry of nanomedicine into tumor tissue through the EPR effect. The nanomedicines diffuse through the permeable tumor blood vessels and are retained within the tumor interstitium, thus providing a window of opportunity for the extended release of the therapeutic agents directly at the site of the tumor, enhancing treatment efficacy and reducing off-target toxicity.

Passive targeting is heavily dependent on the Enhanced Permeability and Retention (EPR) effect. First coined by Matsumura and Maeda in 1986, the EPR effect is a transport phenomenon defined by the existence of fenestrations in the tumor vascular system. The newly emergent vessels in solid tumor tissues continually hyperplasia with a structurally poor vascular wall and substantial spaces, concomitant with defective lymphatic reflux. This EPR effect promotes the high selectivity, permeability, and retention of nanomedicines, sizes ranging from 10 to 200 nm, allowing for preferential distribution in tumor tissues (Figure 3). The effects, along with reducing toxic side effects, enhance the clinical efficiency of drugs (Wu, 2021). However, research performed by Chan et al. revealed insufficient fenestrations or endothelial spaces in the blood vessel wall to facilitate effective extravasation and nanoparticle accumulation within xenograft tumor models and numerous human tumor specimens. Moreover, prolonged blood circulation failed to transport nanomedicines to the tumor site, with the dense extracellular matrix obstructing the complete nanoparticle penetration into the tumor (Pandit et al., 2020). Consequently, only a few drug molecules can target the tumor site while many were accumulated in organs such as the liver, spleen, and kidneys, hindering nanoparticle accumulation in tumors and potentially inducing toxic side effects (Golombek et al., 2018). To enhance passive targeting efficacy, Shi et al. advocated for combining pharmacological and physical treatments, utilizing the ultrasound thermal effect and the nanomedicine’s nano-effects to re-engineer the tumor microenvironment. The team also outlined the fundamental principles of nanomedicine-based combined treatments and recent clinical advancements in integrating nanomedicine therapy with photodynamics and immunotherapy (Shi et al., 2020). Additionally, the EPR effect is modulated by several *in vivo* factors, including EPR enhancers such as AT-II (vasoconstrictor), peroxynitrite (free radical that affects vascular endothelial integrity), nitroglycerin (nitric oxide-releasing agent), and vascular permeability promoters like bradykinin, prostaglandins, VEGF, and other inflammatory cytokines. These substances enhance the EPR effect by raising vascular permeability and macromolecular extravasation (Li et al., 2017). The EPR effect of nanomedicines has been found to be influenced by factors such as vascular injury, tumor density, and blood flow. Within the tumor tissue, the proliferation of cancer cells creates significant pressure, which compresses the lymphatic and vascular systems. This compression leads to vascular failure and causes the concentration of functional lymphatics and blood vessels on both sides of the tumor. The distribution of vasculature within the tumor is uneven, with greater unevenness from the periphery to the center, further hindering the permeability of nanomedicines (Munir, 2022). To address this issue, there is a need to enhance EPR-based drug delivery by modifying tumor vasculature, angiogenesis, vascular structure, blood flow, etc. Subhan et al. (2023) utilized a combination therapy of angiogenesis inhibitors and chemotherapy to normalize the vascular system and enhance the effectiveness of EPR effect-mediated drug delivery in cancer therapy. Summarily, while the EPR effect poorly promotes nanomedicine uptake by tumour cells and has a relatively inefficient nanomedicine delivery, active targeting can more efficiently deliver nanomedicines to tumour tissues *in vivo*. While the EPR effect is a foundational concept enabling selective nanoparticle accumulation in tumor tissue, its efficacy can be inconsistent due to biological heterogeneity among tumor types and within the tumor microenvironment. Factors such as irregular and dysfunctional tumor vasculature, high interstitial fluid pressure, and the varied expression of permeability factors across tumor types can substantially reduce the passive targeting effectiveness (Karpuz et al., 2020b). These limitations necessitate a deeper understanding and potentially the combination with active targeting strategies to enhance delivery efficiency and therapeutic outcomes. Our discussion now acknowledges these challenges, recognising that a successful translation into clinical practice requires strategies to overcome or complement the EPR effect.

### 4.2 Ligand-mediated active targeting

**FIGURE 4:**
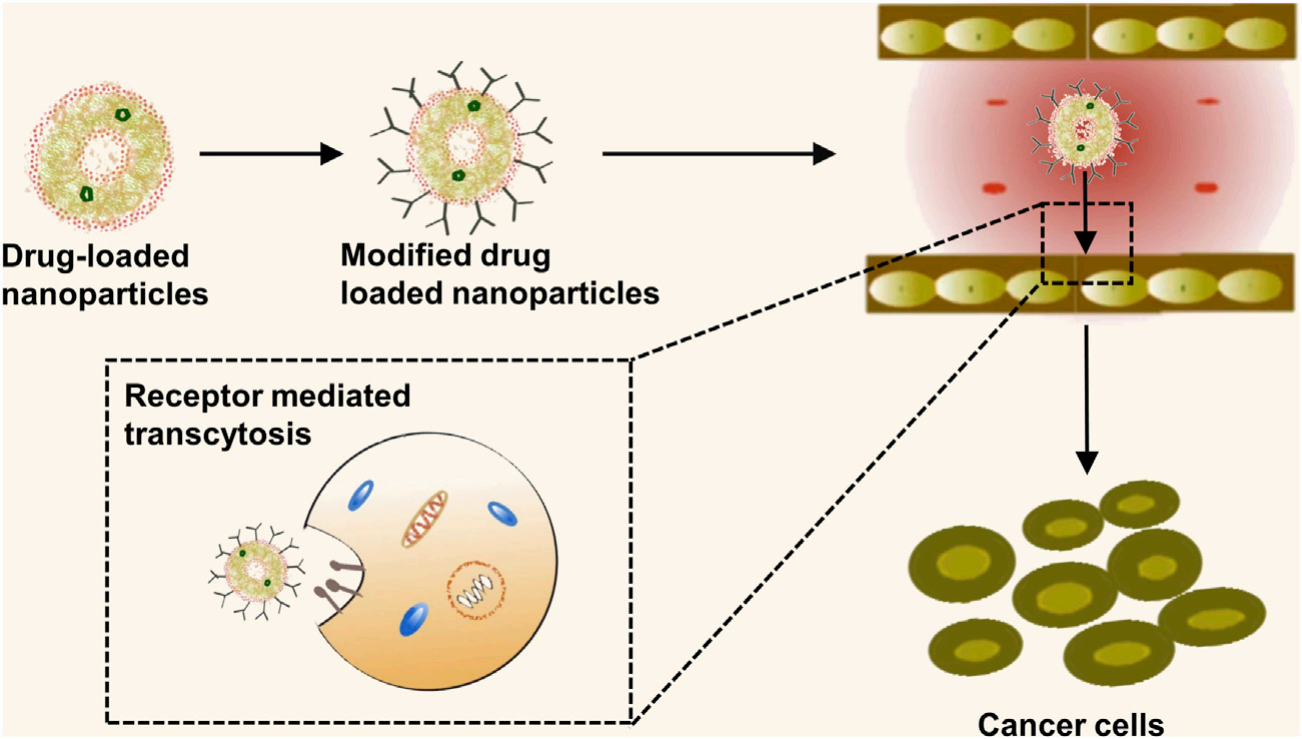
Active targeting mechanisms of nanomedicine. This schematic representation delineates the process of active targeting, whereby the surface of nanomedicine formulations is functionalized with high-affinity ligands. These specialized ligands are engineered to precisely interact with specific receptors overexpressed on the surface of tumor cells.

**FIGURE 5:**
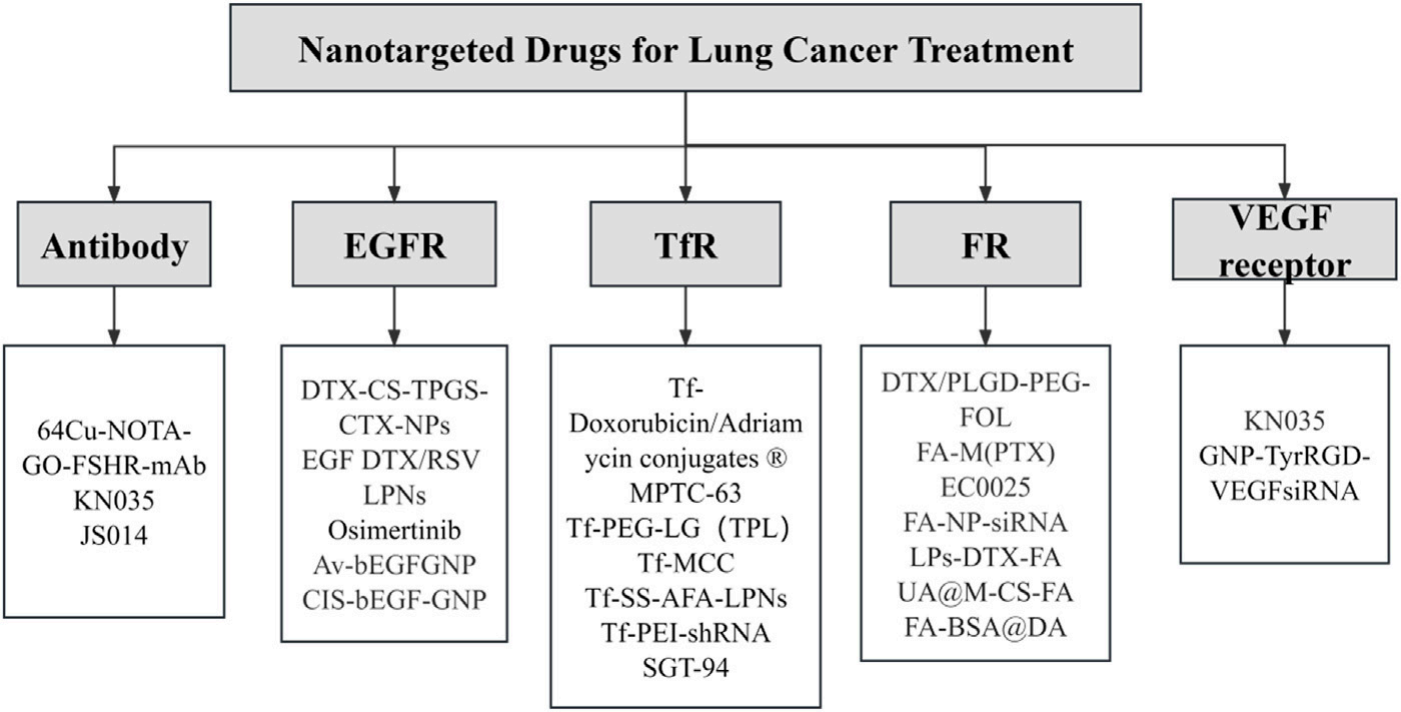
Targeted nanotherapeutics for different targets in lung cancer. This illustration provides an in-depth visual overview of targeted nanotherapeutic strategies and their corresponding molecular targets in lung cancer therapy.

Surface coupling of nanomedicines with high-affinity ligands enables a specific link to the receptor present on the surface of tumor cells, leading to an increase in drug uptake by these cells (Baetke et al., 2015). Additionally, actively targeted nanocarriers for small molecule therapeutics have demonstrated potential in obstructing multiple drug resistance (MDR) through the circumvention of P-glycoprotein-mediated drug expulsion mechanisms (Bazak et al., 2015). Given the relatively low delivery efficiency of passively targeted nanomedicines, active targeting could enhance the delivery efficacy of nanomedicines by modification to bind to specific receptors on the surface of tumor cells. Thus, creating a new therapeutic modality combining passive and active targeting could potentially amplify the drug uptake by tumor cells (Figure 4). However, current applications of active targeting primarily involve the singular use of each therapy target, often yielding unsatisfactory results. The proposition of multi-targeted combination drug delivery might provide a novel direction for the diagnosis and treatment of lung cancer. Therefore, multi-target modification experiments on nanomedicines may reveal their regulatory mechanisms and prospective applications in clinical therapy strategies, forming the theoretical groundwork for the exploration of new anticancer drugs. Several ligands, antibodies, or short peptide-modified nanocarriers, including transferrin, folic acid, and anti-CD47 antibodies, with high specificity and affinities have been developed, considering the specific high expression of receptors or protein antigens on tumor cell membranes (Figure 5) (Muhamad et al., 2018).

#### 4.2.1 Antibody-targeted therapy

**FIGURE 6:**
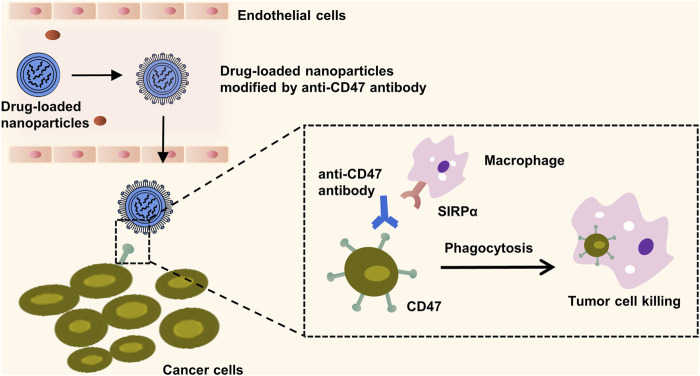
Macrophage mediated phagocytosis enhanced by CD47-targeting nanoparticles. The illustration outlines the mechanism by which the surface modification of nanoparticles with the anti-CD47 antibody competes with CD47 proteins on the cancer cell membranes for binding to the signal regulatory protein alpha (SIRPα) expressed on macrophages. This competitive inhibition prevents the cancer cell’s evasion from macrophage-mediated phagocytosis. Subsequently, the engagement of these modified nanoparticles with SIRPα triggers an immune response that facilitates the engulfment and destruction of the targeted cancer cells.

An antibody, characterised by two epitope binding sites within a single molecule, possesses a small size, extraordinary selectivity, and extremely high binding affinity (Doroudian et al., 2021a). Monoclonal antibodies are promising tools in lung cancer therapy and molecular imaging, with their potential to specifically target overexpressed tissues and efficiently interfere with molecular regulation and tumor signal transduction within these tissues. The first monoclonal antibody capable of binding to specific tumor antigens was developed by Kohler and Milstein in 1975 (Bazak et al., 2015). Presently, monoclonal antibodies such as Cetuximab are widely utilised in clinical practice, demonstrating significant inhibitory effects on the proliferation and growth of NSCLC cells (Bradley et al., 2015). CD47, also referred to as integrally associated protein (IAP), is routinely highly expressed in tumor tissues. It functions to inhibit macrophage phagocytosis by binding to SIRPα, aiding tumor cells in evading immune attack. However, anti-CD47 antibodies can competitively inhibit CD47’s binding to SIRPα and mediate macrophage phagocytosis, thereby targeting and killing tumor cells, such as those found in NSCLC (Theruvath et al., 2022). This presents novel potential treatment approaches for tumors. Currently, antibodies against specific lung cancer targets like anti-EGFR, anti-VEGF, and anti-CD147 are under study (Figure 6) (Theruvath et al., 2022). However, there is a potential downside antibody can provoke an immunogenic response. The latest developments in antibody engineering focus on the design of humanised, chimeric, or fragmented antibodies to attenuate such immunogenic responses (Yu et al., 2012).

Cell-bionic nanoparticles utilize natural cell membranes as the encapsulation medium for nanoparticles (NPs). These nanoparticles have an outer layer composed of natural cell membranes, which provides a protective effect for the interior of the cell-based biomimetic drug delivery system (CDDS). The presence of surface antigens on the cells allows the CDDS to avoid immune recognition by immune cells, leading to a significant prolongation of its circulation time and a reduction in its immunogenicity *in vivo*. The biocompatibility and readily biodegradable nature of the cell membranes contribute to the good biocompatibility and minimal side effects of the CDDS. Additionally, certain cell membranes derived from tumor-growing fractions have the ability to localize cancer-based targets and even cross biological barriers (Zhao et al., 2022). Erythrocyte membrane-biomimetic nanoparticles (RBC-NPs) are the first membrane-encapsulated NPs to incorporate the membrane protein CD47, which is widely expressed on erythrocytes. This incorporation prevents clearance by the immune system by delivering a 'don’t-eat-me' signal, thereby enhancing the targeted delivery of nanoparticles (Dhas et al., 2022). A potent nanosystem based on RBCM-encapsulated MMP2-stimulating peptide binding miR-126-3p has been developed. This novel biomimetic nanoparticle effectively induces apoptosis and inhibits tumor growth and metastasis in cancer cells by targeting ADAM9. So far, various cell membranes, including erythrocyte membranes, macrophage membranes, platelet membranes, leukocyte membranes, and cancer cell membranes, have been utilized in the development of biomimetic nanoparticles (Wu et al., 2022). In order to further integrate multiple functions, research on cellular biomimetic nanoparticles extends beyond individual cell membranes to include hybrid cell membrane-coated nanoparticles (HCMN), which are generated by fusing different cell membranes. These HCMNs are used for targeted cancer therapy (Liu et al., 2023). A prime example of such advances is the study reported by Zheng et al. (2021), which drew inspiration from the cellular cytoskeleton, researchers have engineered a nanomedicine termed Nery-PMIV, which envelops a Au(I)-peptide complex that targets cancer-promoting MDM2/MDMX proteins. Encased in an erythrocyte membrane, Nery-PMIV exhibits increased stability and circulation, effectively enhancing anti-PD1 therapy in lung cancer models and offering a novel, synergistic approach to cancer treatment.

#### 4.2.2 EGFR-targeted therapy

Epidermal growth factor receptor (EGFR), a transmembrane protein equipped with cytoplasmic kinase activity, belongs to the ErbB receptor family. It is expressed in normal cells and responds to a specific epidermal growth factor (EGF) ligand, triggering cell proliferation, survival signaling pathways, and transducing necessary growth factor signals from the extracellular milieu into cells. Critically, EGFR contributes to the accelerated proliferation of tumor cells and promotes angiogenesis, rendering it an optimal target for targeted NSCLC therapy (Zhou et al., 2020). The EGFR family consists of four members: EGFR, HER2, HER3, and HER4. These members have similar structures, with extracellular ligand-binding domains, intracellular tyrosine kinase domains (except for HER3), and intermediate transmembrane structures. Currently, EGFR-targeted drugs primarily include tyrosine kinase inhibitors (TKIs) and monoclonal antibodies (mAb). TKIs work by inhibiting the activation of the EGFR signaling pathway through interaction with the intracellular structural domain, specifically inhibiting the phosphorylation of tyrosine kinase. Common TKIs include first-generation gefitinib and erlotinib, second-generation afatinib and daclatinib, and third-generation ositinib. On the other hand, mAb binds to the extracellular structural domain of the EGFR, preventing ligand binding and blocking the EGFR signaling pathway. Cetuximab (Erbitux) and panitumumab (Vectibix) are both FDA-approved monoclonal antibodies (Hong et al., 2023). However, EGFR TKIs are not effective against advanced mutant non-small cell lung cancer (NSCLC), as it has developed resistance. Cetuximab, a chimeric human-mouse monoclonal antibody, binds competitively with high affinity to EGFR. It inhibits lung cancer cell proliferation, expedites apoptosis, and diminishes angiogenesis, invasion, and metastasis by blocking EGFR signaling (Harding and Burtness, 2005). As Glazer et al. demonstrated, cetuximab is capable of effectively targeting EGFR-positive cancer cell lines *in vitro* (Glazer et al., 2010) and then inducing thermal ablation of malignant cells through a photothermal effect brought about by the heat generated by metal nanoparticles upon exposure to non-ionizing radio-frequency energy (Bazak et al., 2015). The EGFR pathway’s interaction with integrin activates matrix metalloproteinases, modifying cell adhesion, stimulating cell motility and invasion, and promoting tumour metastasis (Harrison et al., 2020). Haikala et al. (2022) developed an antibody-drug coupling (ADC) called patritumab deruxtecan (HER3-DXd; U3-1402) for the treatment of EGFR-mutated NSCLC. This ADC consisted of a human HER3-targeting antibody conjugated to a topoisomerase I inhibitor (DX-8951 derivative or DXd) known as patritumab. The researchers used EGFR inhibitor-resistant PDX models and observed that most of the models expressed both HER2 and HER3, with high prevalence of HER3 expression. This finding suggests that HER3 could be a potential therapeutic target. Furthermore, they discovered that pretreatment with EGFR TKI increased HER3 expression *in vitro* and *in vivo*, leading to enhanced internalization of patritumab deruxtecan and DNA damage response. To enhance clinical relevance, the researchers demonstrated the improved efficacy of patritumab deruxtecan in combination with ositinib using various model systems, offering a novel strategy to overcome EGFR TKI resistance (Lim et al., 2022). Recent studies have shown that dual inhibition of EGFR using EGFR-TKIs and anti-EGFR mAb exhibited strong antitumor activity in patients with EGFR mutation-mediated acquired resistance to TKIs. Combination therapy involving cetuximab/EGFR-TKI has demonstrated clinical efficacy and a manageable safety profile. Additionally, there is ongoing investigation into the anti-tumor activity of cetuximab in combination with NK cell immunotherapy (NCT04872634), which will further advance the treatment of EGFR-mutated NSCLC (Ciardiello et al., 2024). These factors underpin EGFR’s role as an ideal target for lung cancer therapy and intervention, thereby offering fresh perspectives in the development of novel anti-cancer drugs targeting EGFR.

#### 4.2.3 Transferrin receptor-targeted therapy

The transferrin receptor (TfR) is an essential membrane-associated glycoprotein involved in ensuring cellular iron uptake and cell growth regulation (Candelaria et al., 2021). The TfR family encompasses TfR1 (also referred to as CD71) and TfR2, with TfR1 typically expressed at low levels across most healthy human tissues and TfR2 mostly restricted to hepatocytes. Various studies have revealed remarkably upregulated Tf levels in malignant cells compared to normal cells (Candelaria et al., 2021), making the conception of drugs targeting Tf a burgeoning research area in lung cancer therapy (Daniels et al., 2012). Elevated TfR expression in cancer cells serves the increased iron demand, a crucial element for cancer cell growth and proliferation. As such, overexpression of TfR engenders a heightened dependence on iron for these cells and a greater vulnerability to iron deprivation. Consequently, targeting TfR could potentially disrupt the cancer cells' iron supply and inhibit their growth. Iron uptake occurs via TfR interactions that promote the internalization of Tf, with TfR1 being the main pathway for iron-bound Tf to enter the cell and prevent the toxic dispersal of circulating Fe^3+^ in the body. There are two strategies for Tf-targeted therapy: 1) Anti-cancer drugs with high affinity and specificity can bind to Tf, delivering drug molecules to malignant tumours. 2) Molecules antagonizing the normal function of Tf can induce tumour cell death. Xu et al. demonstrated that the delivery of plasmids containing p53 tumour suppressor genes encapsulated in liposomes targeted using anti-transferrin receptor mab, effectively sensitized p53-transfected, radiation-resistant squamous cell cancer cell lines to ionizing radiation. Their results underscored the role of Tf as a ligand in significantly enhancing the transfection efficacy of cationic liposomes (Xu et al., 1999). Zhu et al. harnessed the large TfR expression, reactive oxygen species content, and slightly acidic nature of the lung cancer tumour microenvironment for targeted drug delivery and controlled release using nanomaterials. The elevated expression of TfR in cancer cells is also related to the creation of ribonucleotide reductase during the DNA synthesis of fast-dividing cells (Zhu et al., 2021). Zhao et al. (Zhao X. et al., 2023) developed a new redox-sensitive amylopectin/PTX active drug NPs (PULL-SS-PTX NPs), and further added the cancer cell-targeting ligand transferrin (TF) to its surface, resulting in TF-PULL-SS-PTX NPs. In their experiments, they found that TF-PULL-SS-PTX NPs rapidly decomposed its self-assembled structure under appropriate conditions, triggering drug release with better tumor suppression properties and showing lower systemic toxicity compared with PULL-CC-PTX NPs. Therefore, the iron-assisted production of ribonucleotide reductase may serve as a promising research direction for targeted therapy for lung cancer (Table 4).

**TABLE 4:** Targeted nanomedicines for transferrin receptor.

Nanomedicine	Therapeutic agent	Action mechanism	Advantages	Ref.
Tf-Doxorubicin/Adriamycin conjugates ^®^	DTX	Generation of free radicals and inhibition of certain enzymes (e.g. topoisomerase II)	Reduced toxic effects of adriamycin, saturated with iron or gallium nitrate (GN), with the ability to overcome multidrug-resistant tumor cells	Daniels et al. (2012)
MPTC-63	Platinol-AQ	Leads to intra- or inter-strand cross-linking of DNA, activating multiple signaling pathways and inducing oxidative stress, ultimately leading to tumor cell death	Strong targeting, low toxicity, high temperature and pH tolerance to avoid drug leakage during preparation and storage	Tortorella and Karagiannis (2014)
SGT-94	RB94cDNA	High RB94 protein expression is detected in the cytoplasm of cancer cells by immunochemical staining	Effectively internalized via receptor-mediated endocytosis, well tolerated, for effective delivery of RB94	Siefker-Radtke et al. (2016)
Tf-PEI-shRNA	shRNA	The shRNA is recognized and cleaved by the intracellular nuclease Dicer, generating the RNA-induced targeting complex (RISC), which leads to the degradation of the targeted mRNA or the inhibition of the translational process, thus effectively suppressing the expression of specific genes	Tf binds to the receptor on the cell surface to achieve cell-specific transfection, and PEI, as a transfection vector, is able to efficiently translocate the shRNA guide sequences into the cytoplasm	Liu et al. (2009)
CALAA-01	siRNA	Interferes with tumor growth inhibition to reduce expression of the M2 subunit of ribonucleotide reductase (R2)	Utilizing the guidance function of transferrin, the most effective tumor targeting is achieved through endocytosis mediated by transferrin receptor on the surface of tumor cells into tumor cells	Davis (2009)

#### 4.2.4 Folate receptor-targeted therapy

The folate receptor (FR) is a glycosyl phosphatidylinositol-anchored cell-surface glycoprotein. High FR levels have been detected in tumor cells from NSCLC patients, particularly those with lung adenocarcinoma. Since FR is absent in normal tissues, it can potentially serve as a drug target for targeted therapy in NSCLC (Fan et al., 2023). Folic acid, owing to its high affinity for FR, and FR-mediated cyclic endocytosis can facilitate the internalization of folate-modified drugs into tumor cells. Consequently, coupling folic acid with nanoparticles (NPs) is probable to enhance the efficiency of FR-mediated targeted delivery of therapeutic drugs. Both *in vitro* and *in vivo* experiments corroborate that folic acid modification can bolster the delivery efficiency and safety of nanomedicines while augmenting efficacy of chemotherapy for lung cancer patients (Muhamad et al., 2018). Cagle et al. demonstrated that both lung adenocarcinoma and squamous cell carcinoma exhibit relatively high levels of FR. Further, there was a strong correlation observed between FR expression level and tumor stage as well as survival rates in lung adenocarcinoma (Cagle et al., 2013). Researchers have also demonstrated the successful targeting of FR-positive tumor cells *in vitro* and *in vivo* by combining folate with various therapeutic probes (Shi et al., 2015). The high FR expression on the surface of tumor cells indicates that these cells need folic acid for rapid cell division. Moreover, since FR is abundantly present on tumor cell membranes, it can potentially be exploited as a new molecular target to enhance anti-cancer efficacy (Cagle et al., 2013). Karpuz et al. developed a novel folate-targeted nanodrug-loaded PCX and VNB radiolabeled Tc-99 m for effective imaging and therapy of NSCLC. They conducted *in vitro* tests using LLC 1 cells, C57BL/6 mice, and histopathology studies. The results showed that compared to the combination of free drugs, the use of folic acid targeting and codrug sealing liposomal preparation had several advantages. These advantages included reducing the observed toxicity in single drug therapy, targeting different mechanisms of drug resistance, promoting effective tumor growth, limiting lung metastasis, and enhancing therapeutic response through synergistic or cumulative drug effects. Therefore, folate-targeted combination drugs encapsulated in liposomes hold promise as an agent for effective cancer imaging and therapy. This therapy has the potential to benefit a larger proportion of lung cancer patients (Karpuz et al., 2020a; Karpuz et al., 2021). These combined observations clearly indicate that the FR is a promising therapeutic target, and a larger population of lung cancer patients could potentially benefit from FR-based therapy.

#### 4.2.5 VEGF receptor-targeted therapy

Vascular endothelial growth factor (VEGF) is a unique growth factor specific to vascular endothelial cells. The family of VEGF comprises VEGF-A, VEGF-B, VEGF-C, VEGF-D, VEGF-E, and placental growth factor (PGF). Highly expressed in NSCLC, VEGF drives growth, invasion, and metastasis of lung cancer cells. It induces developmental angiogenesis via the VEGFR-2-dependent signaling pathway by promoting survival and proliferation of vascular endothelial cells (EC) (Adams and Alitalo, 2007). While all VEGF isoforms bind to two receptors, VEGFR-1 and VEGFR-2, VEGF exhibits a ten-fold higher affinity for VEGFR-1 than VEGFR-2 (Apte et al., 2019). Given VEGF’s crucial role in angiogenesis and its conspicuous expression in tumor and stromal cells, particularly in inflammatory cells of human tumors, the first developed angiogenesis inhibitor for clinical use targeted the VEGF signaling pathway [20]. Early experiments discovered that blocking VEGF’s bioavailability using monoclonal antibodies significantly inhibited tumor angiogenesis and growth of various human tumor xenografts in non-immunodeficient mice (Melincovici et al., 2018). Moreover, enhanced VEGF expression is often associated with tumor progression and recurrence. In numerous preclinical models, VEGF-blocking antibodies have displayed significant antitumor activity, escalating the range of tumor growth inhibition between 25% and 95% (Apte et al., 2019). Therefore, by blocking VEGF binding to receptors, the vascular system may be restored to a more “normal” state, decreasing blood vessel density and inhibiting the creation of new blood vessels, thus reducing tumor morbidity and mortality (Lopes-Coelho et al., 2021). However, excessive use of antiangiogenic drugs can have negative effects on drug and oxygen delivery, potentially causing adverse outcomes. Therefore, further research is needed to determine the appropriate selection and dosage of these drugs. Another challenge associated with anti-angiogenic drugs is the development of resistance, which limits their effectiveness as a therapeutic option. Xu et al. (2022) discovered that inhibitors targeting anti-VEGFR2 can lead to upregulation of ADRB2 and subsequent therapeutic resistance to VEGFR2-TKIs. However, blocking the ADRB2/CREB/PSAT1 pathway can enhance the sensitivity of NSCLC to VEGFR2-TKIs, offering a promising combined approach to improve the antitumor efficacy of these drugs. It is important to note that VEGF not only plays a role in angiogenesis, but also acts as an immunomodulator in the tumor microenvironment (TME). VEGFs can inhibit antigen presentation and stimulate the activity of regulatory T cells (Treg) and tumor-associated macrophages, creating an immunosuppressive microenvironment in NSCLC. Therefore, a comprehensive treatment approach for NSCLC should include both VEGF-VEGFR targeted therapy and immunotherapy. Firstly, anti-angiogenic drugs can normalize tumor vasculature and increase the presence of immune cells in NSCLC. Subsequently, immune checkpoint inhibitors can alleviate the suppression of T cells by PD-1 and PD-L1 (Zhao Y. et al., 2022; Patel et al., 2023). Recent studies have demonstrated that the combination of these two approaches yields better therapeutic outcomes for solid tumors, highlighting the potential of combining anti-VEGF drugs with immune checkpoint inhibitors for NSCLC therapy.

## 5 Emerging lung cancer immunotherapy

In recent years, nanoparticle immunotherapy has shown great potential in the therapy of lung cancer. Immunotherapy enhances the immune system to target and eliminate cancer cells, thereby aiding in the treatment of malignant tumors, reducing metastasis, and preventing tumor recurrence (Gowd et al., 2022). Immune checkpoint therapy (ICT) forms the foundation of modern immunotherapy. Immune checkpoint inhibitors (ICIs) have the ability to block inhibitory pathways, suppressing the immune response to cancer and restoring as well as maintaining anti-tumor immunity. Immune checkpoints (ICPs) are proteins produced by certain immune cells (e.g., T cells) and cancer cells, including CTLA-4, PD-1, PD-L1, TIM-3, CD73, and TIGIT (Doroudian et al., 2023). However, only a small proportion of patients respond to these therapies, while the majority do not, leading to drug resistance and tumor recurrence. To enhance the effectiveness of immunotherapy, various techniques are being investigated clinically. These techniques include combining radiotherapy with chemotherapy, combining immunomodulatory drugs with immunotherapy, and combining immunotherapy with other therapies (Gowd et al., 2022). In a study by Reda et al. (2022), a nanoparticle loaded with a PLK1 inhibitor and coupled with a PD-L1 antibody (ARAC) was developed to synergize the effects of PLK1 inhibition and PD-L1 blockade. The aim was to enhance the efficacy of PD-L1 inhibitors. The researchers also discovered that cytotoxic drugs, apart from PLK1 inhibitors, have been found to increase PD-L1 expression. Co-delivering these drugs and PD-L1 antibody with nanoparticle constructs would improve efficacy while reducing toxicity. Another study by Yang et al. (2020) focused on the development of a hyaluronic acid-cisplatin/polystyrene-poly(methylenedicarbazide) (HA-CDDP/PMet) dual-precursor co-assembled nanoparticle for chemo-immunotherapy of lung cancer. The nanoparticles showed increased tumor accumulation, tumor growth inhibition in Lewis lung cancer mice, and prolonged overall survival without nephrotoxicity. Guo et al. (2023) designed a pH-responsive nanomedicine called LRT, which was loaded with a T-type calcium channel inhibitor (TTA-Q6) and a small-molecule inhibitor of CD47 (RRX -001). This nanomedicine was used to activate macrophages and dendritic cells, ultimately reversing immunosuppression in lung tumors. The nanomedicine had a submicrometer-sized, lamellar-structured, and pH-responsive design. In the acidic tumor environment, the release of TTA-Q6 disrupts calcium uptake by cancer cells, leading to endoplasmic reticulum stress. This stress induces the translocation of calcium reticulin to the cell surface. The presence of surface calreticulin activates anti-tumor T cells, which in turn activate macrophages and promote the maturation of dendritic cells. This process enhances effective antigen presentation. Additionally, RRX-001 reduces the levels of CD47 protein, preventing immune escape of calreticulin-rich cancer cells. In a lung tumor model using male mice, this combined approach demonstrated anti-tumor effects and immunity to tumor re-exposure, highlighting its potential for lung cancer immunotherapy.

Therefore, it can be concluded that nanomedicine has the potential to enhance the delivery and effectiveness of lung cancer immunotherapy. By utilizing nanoparticles, it is possible to specifically target cancer cells, minimizing harm to healthy cells and improving drug delivery to the tumor site. Moreover, nanoparticles can be engineered to boost the immune response against cancer cells and facilitate the simultaneous administration of multiple immunotherapeutic drugs. This enables combination therapy that targets various pathways involved in cancer progression. Overall, nanomedicine-based immunotherapy holds significant promise for enhancing the efficacy of lung cancer treatment.

## 6 Summary and outlook

In the panorama of lung cancer prevention, diagnosis, and therapy, a wave of innovative nanomedicines and advanced nano-delivery systems has been catalysed, providing new and optimistic horizons. The clinical potential of nanosystems lies in their precision, their ability to deliver therapeutic agents directly to the cancer cells while sparing healthy tissue. Nanomedicines address the challenges associated with traditional therapeutics, such as drug toxicity and tumor heterogeneity. Yet, while targeted therapy remains at the forefront of lung cancer treatment, the impact of single-mode targeting is often disappointing. Active targeting therapies demonstrating promise, especially when designed to interact with overexpressed receptors on cancer cells, could be limited by a singular point of focus, neglecting the complexity of cancer biology.

Passive targeting strategies, meanwhile, are constrained by low delivery rates. The integration of innovative nanomedicine strategies, particularly those aimed at remodeling the TME to overcome immunosuppression, adds another dimension to the fight against lung cancer. This review proposes a paradigm shift: combining active and passive targeting with multi-target nanocomposite drug construction and multi-modal combination therapy. Such innovative approaches could offer a more comprehensive assault against lung cancer and inject new vigor into nanomedicine applications. Challenges remain as many of these advanced nanomedicines are still undergoing clinical evaluation. One pressing issue is overcoming the biological barriers, those formidable gatekeepers that hinder substantial nanomedicine accumulation at the tumor site. In-depth studies are required to understand nanocarrier distribution *in vivo*, controlled drug release, targeted tissue response, safety, and the unique physical, chemical, and biological properties of nanomedicines influenced by quantum and surface effects.

Addressing these challenges will refine the targeting of nanomedicines, enhance their negotiation of biological barriers, improve the delivery and controlled release of therapeutics, and elucidate the therapeutic and toxic effects linked to their novel properties. If we can harness nanomedicine’s ability to modify the TME and enhance the immune response, we may see a considerable shift in the therapeutic landscape for lung cancer patients who currently do not respond to immunotherapy. With focused inquiry and relentless innovation, we stand on the cusp of a new epoch in lung cancer therapy, with nanomedicine leading the charge. The ultimate goal is a paradigm where nanomedicine strategies not only improve the efficacy of immunotherapy but also broaden the population of lung cancer patients who can benefit from these advanced treatments.
